# PYK2 selectively mediates signals for growth versus differentiation in response to stretch of spontaneously active vascular smooth muscle

**DOI:** 10.14814/phy2.12080

**Published:** 2014-07-17

**Authors:** Anirban Bhattachariya, Karolina M. Turczyńska, Mario Grossi, Ina Nordström, Leonard Buckbinder, Sebastian Albinsson, Per Hellstrand

**Affiliations:** 1 Department of Experimental Medical Science, Lund University, Lund, SE‐22184, Sweden; 2 Pfizer Global Research and Development, Cambridge, 02139, Massachusetts

**Keywords:** Calcium, contraction, ion channel, PF‐4594755, stretch

## Abstract

Stretch of vascular smooth muscle stimulates growth and proliferation as well as contraction and expression of contractile/cytoskeletal proteins, all of which are also regulated by calcium‐dependent signals. We studied the role of the calcium‐ and integrin‐activated proline‐rich tyrosine kinase 2 (PYK2) in stretch‐induced responses of the rat portal vein loaded by a hanging weight ex vivo. PYK2 phosphorylation at Tyr‐402 was increased both by a 10‐min stretch and by organ culture with load over several days. Protein and DNA synthesis were reduced by the novel PYK2 inhibitor PF‐4594755 (0.5–1 *μ*mol/L), while still sensitive to stretch. In 3‐day organ culture, PF‐4594755 caused maintained myogenic spontaneous activity but did not affect contraction in response to high‐K^+^ (60 mmol/L) or to *α*_1_‐adrenergic stimulation by cirazoline. Basal and stretch‐induced PYK2 phosphorylation in culture were inhibited by PF‐4594755, closely mimicking inhibition of non‐voltage‐dependent calcium influx by 2‐APB (30 *μ*mol/L). In contrast, the L‐type calcium channel blocker, nifedipine (1 *μ*mol/L) eliminated stretch‐induced but not basal PYK2 phosphorylation. Stretch‐induced Akt and ERK1/2 phosphorylation was eliminated by PF‐4594755. PYK2 inhibition had no effect on mRNA expression of several smooth muscle markers, and stretch‐sensitive SM22*α* synthesis was preserved. Culture of portal vein with the Ang II inhibitor losartan (1 *μ*mol/L) eliminated stretch sensitivity of PYK2 and Akt phosphorylation, but did not affect mRNA expression of smooth muscle markers. The results suggest that PYK2 signaling functionally distinguishes effects of voltage‐ and non‐voltage‐dependent calcium influx. A small‐molecule inhibitor of PYK2 reduces growth and DNA synthesis but does not affect contractile differentiation of vascular smooth muscle.

## Abstract (precis)

We studied the role of the calcium‐ and integrin‐activated proline‐rich tyrosine kinase 2 (PYK2) in stretch‐induced responses of the rat portal ex vivo. PYK2 phosphorylation at Tyr‐402 was increased both by a 10‐min stretch and by organ culture under stretch for 3 days. Protein and DNA synthesis were reduced by the novel PYK2 inhibitor PF‐4594755 (0.5–1 μmol/L), but contractile differentiation was not affected. Basal and stretch‐induced PYK2 phosphorylation in culture were inhibited by PF‐4594755, closely mimicking inhibition of non‐voltage‐dependent calcium influx by 2‐APB (30 μmol/L). In contrast, the L‐type calcium channel blocker, nifedipine (1 μmol/L) eliminated stretch‐induced but not basal PYK2 phosphorylation. The results suggest that PYK2 signaling functionally distinguishes effects of voltage‐ and non‐voltage‐dependent calcium influx.

## Introduction

Vascular smooth muscle cells play dual roles in the vessel wall by, on one hand, regulating blood flow and, on the other, preserving the integrity of the vessel wall via proliferation, migration, and protein synthesis in response to environmental challenges such as mechanical stimuli, tissue injury, and inflammation. This dual role of smooth muscle is reflected in phenotypic plasticity enabling vascular smooth muscle to take on either a “contractile” or a “synthetic” phenotype appropriate for these different roles (Owens 1995). Sustained elevation of transmural pressure across the vascular wall stimulates remodeling of myogenically active resistance vessels by causing constriction and eventually hypertrophy of the vessel wall with ensuing increase of peripheral resistance (Aalkjaer et al. 1987). This sequence of events involves both growth and contractility and cannot be described merely as switching between contractile and synthetic phenotypes. Most large vessels do not develop myogenic tone, but an exception is the rodent portal vein, which has a well‐characterized myogenic tone and rapidly hypertrophies under increased pressure in vivo as well as ex vivo (Malmqvist and Arner 1990; Zeidan et al. 2000). Studies of this vessel have therefore contributed to the general understanding of electrophysiology, contractility, and remodeling in smooth muscle (Albinsson et al. 2014). Here, we used the rat portal vein to elucidate signal mechanisms mediating mechanically induced remodeling.

Recent studies have largely clarified the mechanisms regulating the expression of contractile and cytoskeletal proteins, which is generally driven by serum response factor (SRF) in concert with the coactivators myocardin and myocardin‐related transcription factors (MRTF, a.k.a. MAL). A key element is translocation of MRTF from the cytosol to the nucleus in response to decreased concentration of globular (G‐) actin (Miralles et al. 2003). Conditions favoring polymerization to filamentous (F‐) actin are thus expected to also stimulate the synthesis of smooth muscle proteins and thereby maintenance of the contractile phenotype.

Stretch of the portal vein causes increased overall protein synthesis and increased expression of smooth muscle marker proteins, such as the actin‐binding proteins SM22*α* and calponin and the intermediate filament protein desmin, which are regulated by SRF, myocardin, and MRTF. This is associated with activation of ERK1/2 as well as Rho and actin polymerization (Zeidan et al. 2000, 2003; Albinsson et al. 2004).

Using organ culture of rat and mouse portal vein, we have previously demonstrated that actin polymerization in vascular smooth muscle is stimulated by stretch of the vessel wall, which correlates with synthesis of SRF‐dependent smooth muscle marker proteins (Albinsson et al. 2004). Rho/Rho kinase activation by stretch was found to be instrumental for this effect, and simultaneous work on aortic cells showed that calcium influx via voltage‐dependent (L‐type) channels activates smooth muscle marker expression via Rho/Rho kinase ‐dependent myocardin expression (Wamhoff et al. 2004). Using the portal vein model we subsequently showed that stretch‐dependent smooth muscle marker expression is inhibited by the L‐type calcium channel blocker verapamil and that the effect of calcium influx on myocardin expression is mediated via the transcription factor MEF2 (Ren et al. 2010). In contrast to contractile protein expression, growth and proliferation stimulated by the MAPK pathway and immediate early gene expression was found to be inhibited by the non‐voltage‐dependent calcium influx blocker 2‐aminoethoxydiphenyl borate (2‐APB). This suggests functional compartmentation of calcium signals with respect to regulation of differentiation and growth, respectively.

The myogenic tone of the portal vein is associated with intracellular calcium transients, dependent on L‐type calcium channel activity, and is enhanced by stretch (Johansson and Mellander 1975; Swärd et al. 1993; Spencer and Greenwood 2003). The mechanisms whereby stretch activates the Rho/Rho kinase and MAPK pathways are, however, incompletely known, and in particular the possible role of different modes of calcium influx from the extracellular space is obscure. The FAK‐family nonreceptor tyrosine kinase PYK2 (proline‐rich tyrosine kinase 2, a.k.a. FAK2, CAK*β*, or RAFTK) is known to be phosphorylated in response to multiple stimulants including angiotensin II (Ang II), increased intracellular calcium, and integrin activation, and to influence multiple signal systems including the MAP kinase and Rho pathways as well as ion channel activity (Avraham et al. 2000). This raises the possibility that PYK2 plays a role in the compartmentation of calcium‐dependent signaling for growth versus differentiation and that it is involved in mechanically induced responses. We therefore investigated its possible involvement in stretch‐mediated signaling in the intact rat portal vein, using as a tool the novel selective PYK2 inhibitor PF‐4594755 (Bonnette et al. 2010).

## Methods

### Vessel mounting and incubation

All experiments were approved by the Malmö/Lund animal ethics committee (M167‐09 and M213‐12). This investigation conforms to Directive 2010/63/EU of the European Parliament. Rats (Sprague‐Dawley, 200 g) were bought from Taconic (Denmark). They were sacrificed using CO_2_ inhalation and the portal veins were removed and cleaned from surrounding fatty tissue. The portal veins, which have predominantly longitudinal muscle, were divided longitudinally into two strips and mounted in centrifuge tubes with one strip under stress by a hanging weight (0.6 g, giving optimal load for force development) and the other strip unloaded. The strips were cultured in DMEM‐Hank's F12 (1:1) with 2% dialyzed FCS and 10^−9^ mol/L insulin as described (Zeidan et al. 2000). For short‐term loading, the weight was suspended during overnight incubation to allow vessels to accommodate after mounting, and then released for 10 min before the strip was frozen in liquid nitrogen. Long‐term responses were studied by organ culture under continuous load for 2 days for mRNA analysis by quantitative PCR, and for 3 or 5 days for protein analysis by western blotting. The unloaded control strips were treated identically.

### Chemicals

The PYK2 inhibitor PF‐4594755 (gift from Pfizer Inc., Peapack, NJ) has been described, was dissolved in DMSO, and used at 0.5 or 1 *μ*mol/L (max. 0.08% DMSO), a concentration range showing high kinome selectivity (Bonnette et al. 2010). Vehicle was added to all control preparations. The *α*_1_‐adrenergic receptor agonist cirazoline hydrochloride, the phosphatase inhibitor calyculin A, and the Ang II type 1 receptor inhibitor losartan were purchased from Tocris Bioscience. Nifedipine was purchased from Sigma and 2‐APB from Calbiochem.

### Force recording

Following organ culture under load in the presence or absence of PF‐4594755, portal vein strips were mounted in a myograph (610M, Danish Myo Technology, Aarhus, Denmark) in HEPES‐buffered medium (in mmol/L: 135.5 NaCl, 5.9 KCl, 2.5 CaCl_2_, 1.2 MgCl_2_, 11.6 HEPES; pH 7.4) and maintained at 37°C. Inhibitor or vehicle was added to the medium as appropriate. Vessels were equilibrated for 45 min after applying a basal tension of 3 mN. Following recording of spontaneous activity, strips were contracted twice with 60 mmol/L KCl‐containing HEPES medium. Following each contraction, they were relaxed in normal HEPES medium. They were then contracted with increasing concentrations of cirazoline. Contractile responses were measured by calculating the mean force over a 5‐min interval following addition of agonist (LabChart 7, ADInstruments, Dunedin, New Zealand). Even though the pattern of frequency and amplitude of contractions may vary between preparations and after agonist addition, mean force provides a reliable and reproducible quantitation of spontaneous activity in the portal vein. To induce maximal contractions by irreversible myosin light chain phosphorylation, calyculin A (1 *μ*mol/L) was added in calcium‐free HEPES medium at the end of the experimental protocol.

### Quantitative PCR

Portal veins were quickly frozen in liquid nitrogen and total RNA was isolated using microRNeasy Mini Kit 217004 (Qiagen, Hilden, Germany) according to the manufacturer's instructions. The relative expression of mRNA was analyzed by quantitative PCR (StepOnePlus qPCR cycler, Applied Biosystems, Carlsbad, CA) using QuantiFast SYBR Green RT‐PCR Kit (Qiagen, 204156). The following QuantiTect primer assays (Qiagen) were used: GAPDH (Rn_Gapd, QT00199633), Tagln (Rn_Tagln, QT00188769), Des (Rn_Des, QT01082879), Acta2 (Rn_Acta2, QT01615901), Tpm1 (Rn_Tpm1, QT00194264). Primers sequences are proprietary of Qiagen.

### Protein extraction and western blotting

Proteins were extracted as described previously (Albinsson et al. 2004). Protein concentration was determined with the Lowry method (BioRad reagents, Hercules, CA) prior to sample loading onto the gel. Equal amounts (15–30 *μ*g) of protein were loaded in each lane of Bio‐Rad TGX 4–15% Criterion gels. Proteins were then transferred to a nitrocellulose membrane (0.45 *μ*m pore size, BioRad) either using a semidry transfer (Trans‐Blot® Turbo™ Transfer System, Bio‐Rad) or wet transfer overnight (Bio‐Rad). Proteins were detected using commercially available primary antibodies: Akt (#9272, 1:1000), phospho‐Akt (Ser473, #9271, 1:500), ERK1/2 (p44/42 MAPK, #9102, 1:1000), phospho‐ERK1/2 (phospho‐p44/42 MAPK, #4370, 1:1000), FAK (#3285, 1:1000), purchased from Cell Signaling Technology (Danvers, MA), phospho‐FAK (Tyr397, 44‐624G) and phospho‐PYK2 (Tyr402, #44‐618G, 1:1000) from Invitrogen (Carlsbad, CA), and PYK2 (CAK*β*, P3902, 1:1000) from Sigma (St. Louis, MO). Secondary mouse or rabbit HRP‐conjugated antibodies (#7074, #7076 1:5000 or 1:10,000, Cell Signaling) were used. Bands were visualized using ECL (Pierce West Femto) and images were acquired using the Odyssey Fc Imager (LI‐COR Biosciences, Lincoln, NE).

### Autoradiography

Protein synthesis rates were determined using autoradiography of portal vein strips exposed to [^35^S]‐methionine (PerkinElmer) in low‐methionine medium for the last 24 h of 3–5 day organ culture as described previously (Zeidan et al. 2003). Following electrophoresis, gels were silver stained, dried, and exposed to film at −80°C. The film was developed, scanned, and analyzed using Quantity One software (Bio‐Rad).

### DNA synthesis

After 3 days of organ culture portal vein strips were exposed for 1 h to radio‐labeled methyl‐[^3^H]‐thymidine (1 *μ*Ci/mL; PerkinElmer, Waltham, MA). Following thorough wash with cold PBS, they were then incubated with 10% TCA for 1 h at 4°C to allow unincorporated [^3^H]‐thymidine to leak out. They were subsequently washed twice with TCA and frozen in liquid nitrogen. Radioactivity was measured by liquid scintillation counting. Radioactivity was normalized to the total protein concentration in each sample.

### Statistics

Values are presented as mean ± SEM unless otherwise stated. *P*‐values were calculated by Student's *t*‐test for single comparisons and with two‐way analysis of variance (ANOVA) followed by Bonferroni post hoc testing for multiple comparisons involving effects of both stretch and added inhibitors. Statistical analysis was performed using GraphPad Prism 5 (GraphPad Software Inc.). *P *<**0.05 was considered statistically significant.

## Results

**Figure 1.**
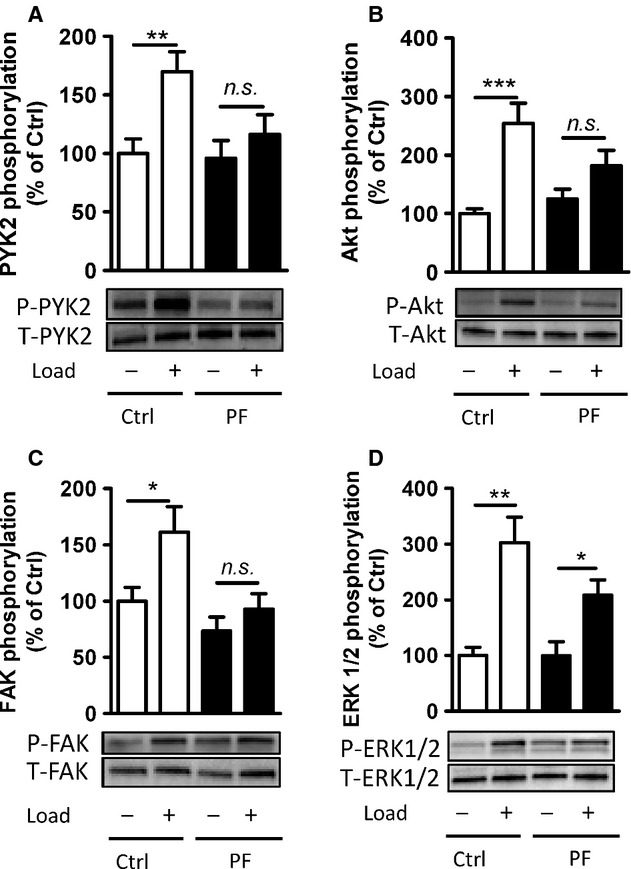
Proline‐rich tyrosine kinase 2 phosphorylation induced by acute stretch is inhibited by PF‐4594755 (PF). Rat portal veins were cultured with either DMSO (0.08%) or PF (1 *μ*mol/L) overnight (A–D). Following overnight equilibration, portal vein strips were stretched by a hanging weight for 10 min and quickly frozen in liquid nitrogen. Phosphorylation of PYK2 (A), Akt (B), FAK (C), and ERK1/2 (D) was determined with phospho‐specific antibodies and the signal intensity normalized to the signal intensity of the respective total protein (*n* = 7–10). *P <**0.05, **P <**0.01, ***P <**0.001, n.s., not significant, for comparison with unloaded strips.

Proline‐rich tyrosine kinase 2 is a cytoplasmic tyrosine kinase that exerts effects on multiple signal pathways, including those regulated by G‐protein‐coupled receptors, MAPK and phosphoinositide (PI) 3‐kinase (Avraham et al. 2000). The effect of stretch on PYK2 phosphorylation (Tyr‐402) and some of its downstream signals was investigated in longitudinal strips of rat portal vein. ERK1/2, a mediator of growth signals downstream of tyrosine kinases, is highly sensitive to stretch and easily activated during dissection and handling of vascular preparations (Zeidan et al. 2000). To ensure basal ERK1/2 phosphorylation, portal venous strips were kept in organ culture overnight before being challenged by stretch. Application for 10 min of a load giving optimal distension for active force generation caused increase in PYK2 as well as Akt, FAK, and ERK1/2 phosphorylation relative to unloaded strips (Fig. 1). To test the role of PYK2 in the downstream signaling pathways we used the novel inhibitor PF‐4594755, which is highly specific for PYK2 at concentrations of 0.5–1 *μ*mol/L (Bonnette et al. 2010). PV strips exposed to 1 *μ*mol/L PF‐494755 were incubated and stretched in parallel with untreated control preparations. As shown in Figure 1, overnight incubation with PF‐4594755 abolished the PYK2, Akt, and FAK phosphorylation in response to 10‐min stretch, while there was still a significant increase in ERK1/2 phosphorylation.

**Figure 2.**
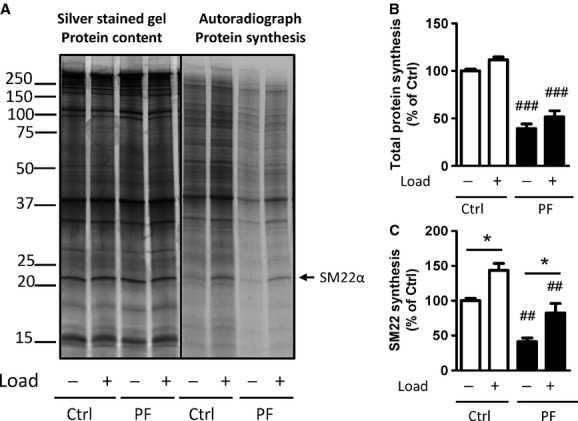
PF‐4594755 reduces protein and SM22*α* synthesis in cultured portal vein. Unloaded/loaded portal veins were incubated in organ culture for 5 days with DMSO (0.08%) or PF (1 *μ*mol/L). [^35^S]‐methionine was added for the last 24 h (A–C). Proteins were separated by SDS‐PAGE and the total amount was visualized using silver staining (A, *left panel*). [^35^S]‐methionine incorporation was visualized by autoradiography (A, *right panel*). Summarized data of total protein (B) and SM22*α* (C) synthesis (*n* = 3). *P <**0.05 for comparison with unloaded strips, ^##^*P *<**0.01, ^###^*P *<**0.001 for comparison with untreated controls (unloaded/loaded).

To investigate long‐term effects of PYK2 on protein synthesis, loaded portal venous strips and unloaded control strips were incubated in organ culture over several days with or without PF‐4594755 and [^35^S]‐methionine incorporation determined by autoradiography of silver‐stained gels loaded with equal protein amounts for all samples. Total incorporation in each lane was used as a measure of global protein synthesis, whereas incorporation in the band representing SM22*α* was used to estimate synthesis of smooth muscle marker proteins. This band is readily visible on silver‐stained gels and has been identified using western blot and mass spectroscopy (Zeidan et al. 2003; Albinsson et al. 2004). [^35^S]‐methionine incorporation values were normalized to the corresponding bands on the silver‐stained gels. Maximal load‐sensitive SM22*α* synthesis appears at around 3 days of culture, but at this time only a small (<10%) load‐induced increase in SM22*α* contents is seen (Zeidan et al. 2003). In initial experiments with PF‐4594755 we therefore extended the culture time to 5 days in an attempt to disclose effects at the protein level. Incubation with 1 *μ*mol/L PF‐4594755 caused profound inhibition of protein synthesis as shown by autoradiography, whereas on the silver‐stained gels there was no appreciable effect on protein composition (Fig. 2A). In this time frame, any decrease in total protein contents due to PYK2 inhibition is therefore not associated with altered relative contents of smooth muscle marker proteins, as exemplified by SM22*α*. Global protein and SM22*α* synthesis are both sensitive to stretch, although the stretch sensitivity of SM22*α* synthesis is relatively more prominent (Ren et al. 2010). PYK2 inhibition dramatically reduced both overall and SM22*α* protein synthesis, but stretch sensitivity persisted (Fig. 2B,C).

**Figure 3.**
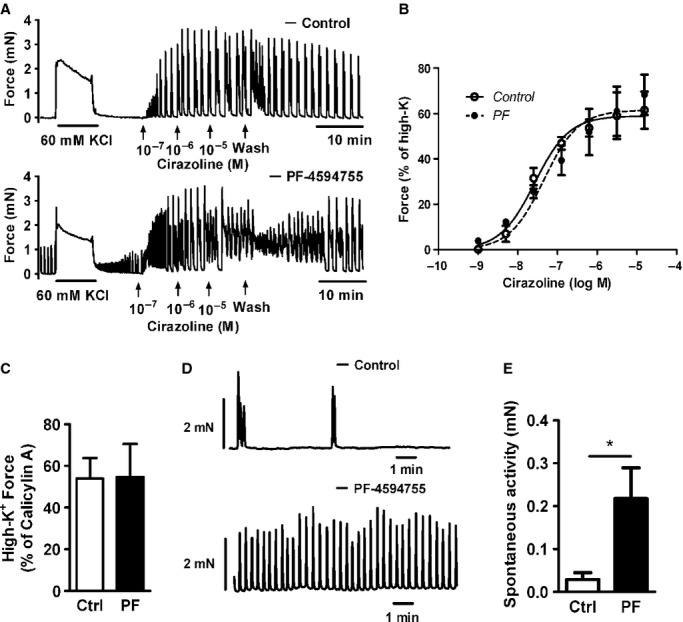
Contractility of portal vein cultured with PF‐4594755. Rat portal veins were cultured either with DMSO (0.08%, control) or PF‐4594755 (1 *μ*mol/L) for 3 days under stretched (loaded) conditions (A–E). After culture, isometric contractile force was evaluated by myography. Representative recordings of force induced by 60 mmol/L KCl‐containing HEPES buffer (high‐K^+^), cirazoline and spontaneous activity in control and PF‐4594755‐treated portal vein strips (A). Concentration–response curve showing mean force in response to cirazoline (B). Maximum response (Control: 59%, PF: 61% of high‐K^+^ force, basal force subtracted) and log EC_50_ (Control −7.57:, PF: −7.34) did not differ (B; *n* = 3). High‐K^+^ force evaluated relative to force induced by the phosphatase inhibitor Calicylin A (C; *n* = 3). Representative recordings (D) and summary of mean force in spontaneous activity (F) in control and PF‐treated portal vein strips (*n* = 6). *P <**0.05.

To test the effect of PYK2 inhibition on contractile responses, portal venous strips were cultured for 3 days under continuous longitudinal stretch in the presence and absence of PF‐4594755 (1 *μ*mol/L). Responses to 60 mmol/L KCl‐containing HEPES buffer (high‐K^+^) and to *α*_1_‐adrenergic receptor stimulation by cirazoline, measured as mean force over 5 min after the addition of agonist (see Methods), were similar in control strips and in strips cultured with PF‐4594755 (Fig. 3A,B). Likewise, the response relative to maximal activation by the membrane‐permeable phosphatase inhibitor calyculin A was the same in control and inhibitor‐treated strips (Fig. 3C). Interestingly, while control strips showed a low frequency of spontaneous activity after organ culture, the spontaneous activity of strips cultured with PF‐4594755 showed higher frequency and mean force, similar to the pattern seen in freshly dissected vessels (Johansson and Mellander 1975; Swärd et al. 1993) (Fig. 3D–E). PF‐4594755 added acutely to PV strips had no effect on either spontaneous activity or responses to *α*_1_‐adrenergic stimulation (data not shown).

Much evidence indicates that stretch/pressure increases the intracellular calcium concentration in smooth muscle cells, which is a basis for the myogenic response (Walsh and Cole 2013). The exact mechanisms probably differ between vascular segments, although an initial depolarization elicited by nonselective, stretch‐sensitive channels is thought to depolarize the membrane sufficiently to activate voltage‐dependent (L‐type) calcium channels, hence stimulating calcium influx and possibly release from intracellular stores (Knot and Nelson 1998). In the portal vein, inhibition of L‐type channels reduces stretch‐induced expression of smooth muscle genes such as SM22*α* and desmin, whereas inhibition of non‐voltage‐dependent calcium influx by the nonselective blocker 2‐APB reduces overall protein synthesis but has no effect on SM22*α* (Ren et al. 2010). We considered that PYK2 may have a role in this apparent selectivity of calcium signaling. Hence, we investigated the effect of PYK2 inhibition on protein synthesis in the portal vein by comparison with those of nifedipine and 2‐APB.

**Figure 4.**
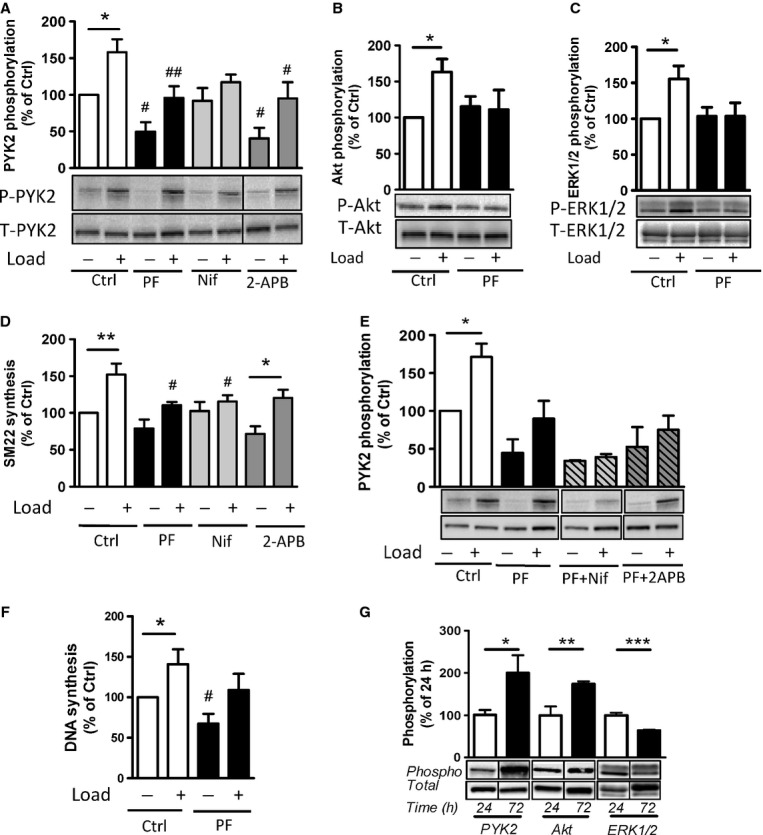
Interactions between PF‐4594755 and inhibitors of voltage/non‐voltage‐dependent Ca^2+^ entry. Portal vein strips were incubated in organ culture with or without load for 3 days in the presence of [^35^S]‐methionine for the last 24 h (A–D). Effect of PF (0.5 *μ*mol/L), nifedipine (Nif, 1 *μ*mol/L), and 2‐APB (30 *μ*mol/L) on PYK2 phosphorylation (A), and effect of PF on Akt (B) and ERK1/2 (C) phosphorylation was evaluated by western blot (*n* = 5–6). Summarized data of SM22*α* synthesis as measured by autoradiography (D; *n* = 6). Effects of the combined action of PF and nifedipine or 2‐APB on PYK2 phosphorylation compared with that of PF alone, normalized to phosphorylation of untreated control as in panel A (E; *n* = 3–4). DNA synthesis as measured by [^3^H]‐thymidine incorporation in portal veins cultured unloaded/loaded for 3 days with or without PF (F; *n* = 6). PYK2, Akt and ERK1/2 phosphorylation of portal vein cultured unloaded for 24 h or 72 h as indicated. (F, *n* = 5). In each panel, all blots shown are from the same gels for phosphorylated or total protein, respectively. *P <**0.05, **P <**0.01, ***P <**0.001 for comparison with unloaded strip, ^#^*P *<**0.05, ^##^*P *<**0.01 for comparison with untreated control (unloaded/loaded).

Given the dramatic effects of 1 *μ*mol/L PF‐4594755 on protein synthesis rates, we reduced the concentration of the inhibitor for comparison with the effects of calcium influx inhibitors. Figure 4 shows effects of PF‐4594755 (0.5 *μ*mol/L), 2‐APB (30 *μ*mol/L), and nifedipine (1 *μ*mol/L) on PYK2 phosphorylation (panel A) and SM22*α* synthesis (panel D) in portal venous strips cultured for 3 days in the absence or presence of longitudinal load. PF‐4594755 and 2‐APB reduced PYK2 phosphorylation in both unloaded and loaded strips, but loaded strips still showed higher PYK2 phosphorylation. However, nifedipine selectively reduced PYK2 phosphorylation in loaded strips. Similar effects were seen on SM22*α* synthesis. Overall protein synthesis was significantly increased by stretch and was reduced in both stretched and unstretched strips by PF‐4594755 and 2‐APB, but only in stretched strips by nifedipine (data not shown). Therefore, relative to overall protein synthesis, neither PF‐4594755 nor 2‐APB had any effect on SM22*α* synthesis, while nifedipine selectively reduced its stretch sensitivity. Taken together, the effects of PF‐4594755 on PYK2 phosphorylation and protein synthesis are similar to those of 2‐APB but differ from those of nifedipine. This is further supported by the evidence that the combined action of PF‐4594755 and 2‐APB did not differ from that of PF‐4594755 alone, while the combination of PF‐4594755 and nifedipine reduced PYK2 phosphorylation to the same low level in both loaded and unloaded strips (Fig. 4E). Stretch‐sensitive signaling via the Akt and ERK1/2 pathways was inhibited following 3 days of incubation with PF‐4594755 (Fig. 4B,C). The stretch‐sensitive mechanism regulating PYK2 and downstream signaling under these conditions likely involves voltage‐dependent calcium influx, since stretch sensitivity of Akt and ERK1/2 phosphorylation was eliminated by nifedipine but not 2‐APB (data not shown), similar to PYK2 (Fig. 4A).

Deoxyribonucleic acid synthesis as determined by [^3^ H]‐thymidine incorporation was inhibited by PF‐4594755 (Fig. 4E). The intact portal vein is kept in an essentially quiescent state during organ culture and load‐induced growth is predominantly hypertrophy. At the early stages investigated here increased DNA synthesis may primarily represent increased DNA contents per cell rather than cell proliferation (Zeidan et al. 2000).

An interesting observation is that PF‐4594755 reduced PYK2 phosphorylation of unloaded as well as loaded strips after 3 days of organ culture (Fig. 4A), whereas after overnight incubation only loaded strips were affected (Fig. 1A). A possible reason for this is that organ culture *per se* increases PYK2 phosphorylation. To investigate this, unloaded portal vein was cultured for either 24 or 72 h and then blotted for PYK2, Akt, and ERK1/2 phosphorylation. As seen in Figure 4D, culture for 72 h significantly increased both PYK2 and Akt phosphorylation, while ERK1/2 phosphorylation was decreased.

**Figure 5.**
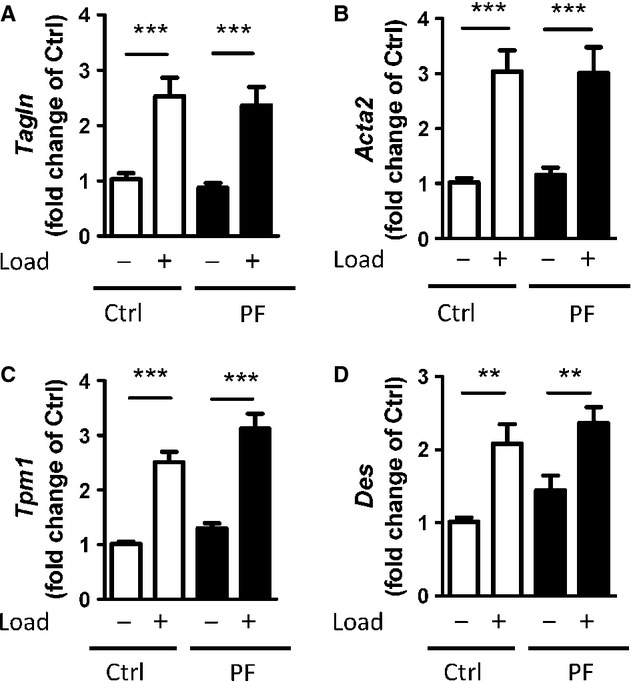
Treatment with the PYK2 inhibitor PF‐4594755 does not alter smooth muscle marker gene expression. Unloaded/loaded portal veins were cultured for 48 h with or without PF. Relative mRNA expression of smooth muscle marker genes was determined by quantitative PCR (A–D): SM22*α* (*Tagln*; A), *α*‐actin (*Acta2*; B), *α*‐tropomyosin (*Tpm1*; C), and desmin (*Des*; D). The mRNA expression level of the target genes was normalized to GAPDH as a reference gene *n* = 6 for all figures. **P <**0.01, ***P <**0.001 for comparison with unloaded strips.

As seen from the results with nifedipine (Fig. 4A) there is an effect of voltage‐dependent ion channel activity on PYK2 phosphorylation, which becomes evident when the channel activity is increased by stretch. This correlates with the role of L‐type calcium channel activity in regulating smooth muscle marker genes via myocardin expression and actin polymerization (Wamhoff et al. 2004; Ren et al. 2010). To examine the role of PYK2 in this response we determined the mRNA expression of several smooth muscle markers following 48‐h incubation of portal venous strips in the presence of 1 *μ*mol/L PF‐4594755 (Fig. 5A–D). There was, however, no effect of the PYK2 inhibitor on the mRNA expression of either SM22*α* (*Tagln*), smooth muscle *α*‐actin (*Acta2*), *α*‐tropomyosin (*Tpm1*) or desmin (*Des*). In particular, the activation of gene expression by stretch was unaffected by the PYK2 inhibitor, as was the basal gene expression. This is similar to the lack of effect of 2‐APB on stretch‐activated smooth muscle gene expression but contrasts with the inhibition due to block of voltage‐dependent calcium channel activity (Ren et al. 2010).

**Figure 6.**
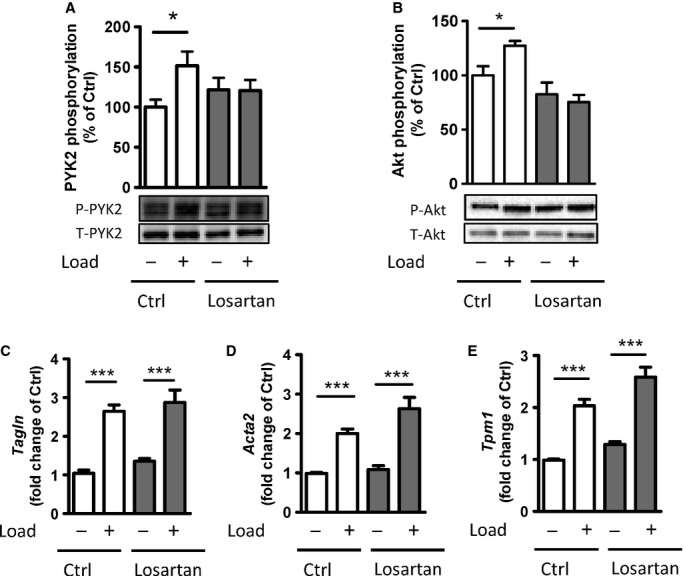
AngII type 1 receptor inhibition eliminates stretch‐induced activation of PYK2 and Akt but not of smooth muscle marker expression. (A–B), Unloaded/loaded portal veins were incubated in organ culture with or without Losartan (1 *μ*mol/L) for 3 days. The phosphorylation of PYK2 (A) and Akt (B) was measured by western blot with phospho‐specific antibodies. The relative mRNA expression of smooth muscle marker genes in portal vein after 48 h of organ culture was analyzed by quantitative PCR (C‐E): SM22*α* (*Tagln*; C), *α*‐actin (*Acta2*; D), and *α*‐tropomyosin (*Tpm1*; E. The mRNA expression level of the target genes was normalized to GAPDH. *n* = 6–7. *P <**0.01, **P <**0.01, ***P <**0.001 for comparison with unloaded strips.

Stretch of the vascular wall has been shown to activate angiotensin II type 1 receptors (AT_1_R), likely via ligand‐independent activation (Mederos y Schnitzler et al. 2008). Since PYK2 is a known mediator of AT_1_R signaling (Sabri et al. 1998), we investigated their interaction in stretch responses in the portal vein. Culture for 3 days in the presence of the AT_1_R inhibitor losartan (1 *μ*mol/L) eliminated the stretch sensitivity of PYK2 and Akt phosphorylation but did not affect their basal levels (Fig. 6A,B). Similar to PF‐4594755, incubation of portal venous strips with losartan for 48 h did not reduce the mRNA expression of the smooth muscle markers SM22*α* (*Tagln*), smooth muscle *α*‐actin (*Acta2*) and *α*‐tropomyosin (*Tpm1*) (Fig. 6D–F).

## Discussion

The effect of stretch on smooth muscle growth and differentiation likely involves both integrin activation and increase of intracellular calcium. Among possible mediators of the response, PYK2 is interesting because of its activation both by integrins and calcium, its association with Src, G‐protein, and MAPK signaling and its interaction with L‐type calcium channels (Lev et al. 1995; Dikic et al. 1996; Avraham et al. 2000). PYK2 has emerged as an interesting therapeutic target both for cancer and for osteoporosis, since it has the potential to regulate cell proliferation, and also osteoclast and osteoblast function (Buckbinder et al. 2007). This has stimulated efforts to develop PYK2 inhibitors with better selectivity than presently available tyrosine kinase inhibitors (Bonnette et al. 2010). Since bone remodeling is stimulated by mechanical load, and PYK2 is regulated both by integrins and calcium, it may exert partly similar functions in bone and vascular tissue.

Extensive studies have characterized the role of PYK2 with respect to Ang II and growth factor signaling, MAPK activation, cell proliferation, and migration in vascular smooth muscle cells (Sabri et al. 1998; Shen et al. 2010; Perez et al. 2011; Gadepalli et al. 2012). The integrated role of PYK2 in vascular remodeling in response to stretch has been less investigated, although Iwasaki et al. (2003) showed increased PYK2 and ERK1/2 phosphorylation by cyclic stretch of vascular smooth muscle cells. Here, we demonstrate that stretch of the myogenically active portal vein causes increased PYK2 phosphorylation at Tyr‐402. Furthermore, by using the novel PYK2 inhibitor PF‐4594755 in organ culture, we show that long‐term PYK2 inhibition is associated with reduced stretch sensitivity of ERK1/2 and Akt phosphorylation, as well as reduced protein and DNA synthesis. This involves calcium‐dependent signals since the effect on protein synthesis is mimicked by 2‐APB and effects on stretch‐activated ERK1/2 and Akt signaling is mimicked by nifedipine. Interestingly, tyrosine kinase inhibition by herbimycin A and MEK inhibition by PD 98059 both selectively reduce ERK1/2 phosphorylation in stretched but not unstretched portal vein in culture (Zeidan et al. 2000). Thus, ERK1/2 phosphorylation is under the control of stretch‐sensitive tyrosine kinase activity, which on the basis of the present results is likely to involve PYK2.

In contrast to the long‐term effects, short‐term (10 min) stretch activates ERK1/2 despite PYK2 inhibition. We have previously shown that activation of ERK1/2 by acute stretch in portal vein is insensitive to both verapamil and 2‐APB (Ren et al. 2010), and hence this response does not critically depend on calcium influx triggering PYK2 signaling. In the context of growth factor signaling, it has been shown that ERK1/2 activation via IGF‐1 depends on inside‐out activation of integrins involving calcium‐dependent PYK2 activation and association with Src (Sekimoto et al. 2005).

Non‐voltage‐dependent calcium influx, as well as agonist‐stimulated release of calcium from intracellular stores, is considered to be important for regulating growth and proliferation of smooth muscle, whereas calcium influx via voltage‐dependent (L‐type) channels has been shown to have a special role in stimulating smooth muscle differentiation (Wamhoff et al. 2006; Beech 2007). The molecular basis for this apparent specificity of calcium signaling, remains however, essentially unknown. In the present work, we show that inhibition of non‐voltage‐dependent calcium influx using 2‐APB in organ culture produces effects on PYK2 identical to those of PF‐4594755, and that there is no additive effect of the two inhibitors. Also, both inhibitors reduce protein synthesis in loaded as well as unloaded vessels. In contrast, inhibition of voltage‐dependent calcium influx using nifedipine has no effect on basal PYK2 phosphorylation but reduces phosphorylation of stretched vessels to the level in unloaded strips. The combination of nifedipine and PF‐4594755 does, however, reduce PYK2 phosphorylation below the level in unloaded strips and totally eliminates the effect of load. Thus, nifedipine has an action on PYK2 phosphorylation which is additive to that of PF‐4594755 and is revealed here specifically in the response to stretch.

Whether the specificity of calcium‐dependent signals represents different pools of calcium activating growth versus differentiation cannot be directly revealed by the present results. Verapamil‐insensitive but 2‐APB‐sensitive calcium influx after depletion of intracellular stores has been demonstrated in portal venous cells, whereas 2‐APB in contrast to verapamil does not affect force in response to high‐K^+^ depolarization (Ren et al. 2010), suggesting functional specialization depending on calcium influx pathway. We cannot exclude that a higher concentration of PF‐4594755 would have eliminated also the nifedipine‐ and stretch‐sensitive component of PYK2 phosphorylation, but it is interesting that phosphorylation of both Akt and ERK1/2 was insensitive to stretch in organ culture with 0.5 *μ*mol/L PF‐4594755, as with 1 *μ*mol/L nifedipine. This suggests that one component of stretch‐responsive signaling for growth and proliferation has a dependence on L‐type channel activity, whereas growth factor‐coupled signaling primarily may involve non‐voltage‐dependent calcium influx.

A 10‐min stretch was here shown to increase PYK2 phosphorylation in portal venous strips that had been equilibrated overnight without load. This increase was sensitive to PF‐4594755 whereas basal PYK2 phosphorylation was not affected, although the inhibitor had been present during the equilibration. After a 3‐day culture, however, PYK2 phosphorylation was found to be reduced in both loaded and unloaded strips cultured with the inhibitor. A possible explanation for this is that constitutive PYK2 phosphorylation is low in short‐term culture but increases with time, as was seen here to be the case in unloaded portal vein. It is notable that organ culture of vascular smooth muscle, including the portal vein, tends to increase the expression of non‐voltage‐dependent ion channels as well as intracellular calcium release on agonist stimulation (Dreja et al. 2001; Ren et al. 2010). This may represent a slowly occurring shift toward a more synthetic phenotype, which is characterized by decreased expression of voltage‐gated (L‐type) calcium channels and increased expression of non‐voltage‐dependent calcium channels as well as intermediate conductance calcium‐activated K^+^ (K_Ca_ 3.1) channels (Beech 2007). These changes are likely to result in increased intracellular calcium concentration in cells under basal conditions, possibly explaining the increased PYK2 phosphorylation sensitive to PF‐4594755.

Since PYK2 has been shown to mediate calcium‐dependent RhoA activation in response to Ang II in vascular smooth muscle cells (Ying et al. 2009) it may be surprising that in the present work PYK2 inhibition had no effect on the expression of smooth muscle markers. However, our results on smooth muscle marker expression were evaluated by a comparison of stretched versus unstretched vessels, and as noted, PF‐4594755 reduced PYK2 phosphorylation but did not eliminate its stretch sensitivity. A further factor that might contribute to maintained contractile differentiation is related to the recent observations on the role of PYK2 in phenotype regulation reported by Zhang et al. (2012). These authors demonstrated that stimulation of TRPM7 channel expression by Ang II triggers phenotype shift in smooth muscle in mouse ascending aorta by a mechanism involving increased activity of PYK2‐ERK1/2‐Elk 1 signaling. This suppresses smooth muscle differentiation via competition with myocardin/MRTF for SRF binding to promoter sites regulating the expression of smooth muscle marker genes (Wang et al. 2004). Our present results are in accordance with such a mechanism operating in reverse, in that PF‐4594755 inhibited protein and DNA synthesis but not smooth muscle marker expression. In addition, AT_1_ receptor inhibition by losartan produced similar results as PF‐4594755 on PYK2 phosphorylation and gene expression. At the concentrations of PF‐4594755 used here, we did not find significant increase of smooth muscle marker genes. However, an indirect indication that PYK2 inhibition may in fact contribute to maintenance of the smooth muscle in a contractile phenotype is the higher level of spontaneous activity noted after culture with the inhibitor, which may indicate inhibition of phenotype shift during culture. The mechanism behind this effect is not revealed by the present results, but it may be speculated that the increase in basal PYK2 phosphorylation with time in culture may correlate with a phenotype shift involving altered ion channel activity.

Interestingly, Zhang et al. (2012) observed that knockdown of TRPM7 channel inhibited Ang II‐induced stimulation of PYK2 activity and increased smooth muscle marker expression in ascending aorta, whereas in cells from thoracic aorta TRPM7 knockdown was associated with decreased smooth muscle marker expression in response to Ang II. The possible involvement of PYK2 in the latter response is not known, but it is apparent that calcium‐stimulated PYK2 activity can activate both the MAPK and Rho pathways, and that the relative effects may depend on cellular context and mode of stimulation.

Some limitations of this study in relation to its possible pathophysiological relevance need to be addressed. The portal vein is unique in being a large vessel with phasic myogenic activity, which in some respects resembles that of myogenically active microvessels, and it is also highly responsive to increased transmural pressure (Albinsson et al. 2014). Its response to biomechanical forces is therefore of potential interest in the context of arterial remodeling, but although general cell physiological mechanisms may be revealed, studies of the portal vein cannot substitute for direct investigation of the relevant vascular beds in studies of hypertension and vascular disease. A second consideration is that the present results depend on the use of a PYK2 inhibitor which, although improved relative to earlier inhibitors, is not expected to have absolute target specificity. In a screen against a panel of kinases, 1 *μ*mol/L PF‐4594755 was found to inhibit PYK2 to 96% and FAK to 42% (Bonnette et al. 2010). We cannot therefore exclude that some of the effects of PF‐4594755 are due to direct inhibition of FAK and possibly other kinases.

In conclusion, we have shown that PYK2 signaling functionally distinguishes effects of voltage‐dependent and non‐voltage‐dependent calcium influx on stretch‐sensitive smooth muscle growth and differentiation. PYK2 may be interesting for specifically targeting growth processes in phenotypically modified relative to contractile vascular smooth muscle.

## Acknowledgment

We thank Pfizer Inc. for the gift of PF‐4594755.

## Conflict of Interest

Leonard Buckbinder is an employee of Pfizer, Inc. All other authors declare no conflict of interest.
